# Drug Repurposing by Tumor Tissue Editing

**DOI:** 10.3389/fonc.2022.900985

**Published:** 2022-06-24

**Authors:** Florian Lüke, Dennis Christoph Harrer, Pan Pantziarka, Tobias Pukrop, Lina Ghibelli, Christopher Gerner, Albrecht Reichle, Daniel Heudobler

**Affiliations:** ^1^ Department of Internal Medicine III, Hematology and Oncology, University Hospital Regensburg, Regensburg, Germany; ^2^ Division of Personalized Tumor Therapy, Fraunhofer Institute for Toxicology and Experimental Medicine, Regensburg, Germany; ^3^ The George Pantziarka TP53 Trust, London, United Kingdom; ^4^ Bavarian Cancer Research Center (BZKF), University Hospital Regensburg, Regensburg, Germany; ^5^ Department of Biology, University of Rome Tor Vergata, Rome, Italy; ^6^ Department of Analytical Chemistry, Faculty of Chemistry, University of Vienna, Vienna, Austria

**Keywords:** anakoinosis, biomodulation, metronomic chemotherapy, PPAR γ, mTOR, umbrella trial, pioglitazone, molecular diagnostics

## Abstract

The combinatory use of drugs for systemic cancer therapy commonly aims at the direct elimination of tumor cells through induction of apoptosis. An alternative approach becomes the focus of attention if biological changes in tumor tissues following combinatory administration of regulatorily active drugs are considered as a therapeutic aim, e.g., differentiation, transdifferentiation induction, reconstitution of immunosurveillance, the use of alternative cell death mechanisms. Editing of the tumor tissue establishes new biological ‘hallmarks’ as a ‘pressure point’ to attenuate tumor growth. This may be achieved with repurposed, regulatorily active drug combinations, often simultaneously targeting different cell compartments of the tumor tissue. Moreover, tissue editing is paralleled by decisive functional changes in tumor tissues providing novel patterns of target sites for approved drugs. Thus, agents with poor activity in non-edited tissue may reveal new clinically meaningful outcomes. For tissue editing and targeting edited tissue novel requirements concerning drug selection and administration can be summarized according to available clinical and pre-clinical data. Monoactivity is no pre-requisite, but combinatory bio-regulatory activity. The regulatorily active dose may be far below the maximum tolerable dose, and besides inhibitory active drugs stimulatory drug activities may be integrated. Metronomic scheduling often seems to be of advantage. Novel preclinical approaches like functional assays testing drug combinations in tumor tissue are needed to select potential drugs for repurposing. The two-step drug repurposing procedure, namely establishing novel functional systems states in tumor tissues and consecutively providing novel target sites for approved drugs, facilitates the systematic identification of drug activities outside the scope of any original clinical drug approvals.

## Introduction

Drug repurposing is defined as an approach for identifying drug activities outside the scope of the original clinical approvement. Drug reprofiling, repositioning or re-tasking are often used as synonyms (1–3). Pre-clinically, drug repurposing may be studied by the classic sequence, identification of a potential target, model systems and finally clinical trials. The combined experimental and clinically based research process is supported by computational data on transcriptomics, proteomics, and metabolomics, at best synergistically integrated (1). Systematic studies on drug repurposing are possible if concerted therapy approaches aim at inducing biological ‘hallmarks’ in tumor tissues for establishing tumor control or resolution. In metastatic cancer, concerted regulatorily active multi-drug schedules may efficaciously reprogram tumor tissues by ‘editing’, heterotypic cell types organized in multiple tumor systems states, i.e., tumor differentiation, transdifferentiation, reconstitution of immunosurveillance and tumor cell death. A broad diversity of reprogramming techniques summarized under the term anakoinosis induction, are used for editing tumor tissues (4–10). Anakoinosis (*ancient greek for communication*) describes the communicative processes therapeutically induced for reprogramming tumor tissues while exhausting their phenotypic plasticity (11). Tumor tissue editing puts the anakoinotic principle to work in a specific tumor tissue to achieve said biological ‘hallmarks’. The basis for therapeutically meaningful reprogramming techniques are tumor-specific development of non-tumor cell autonomous functions and structures providing a huge, diversified repertoire of often non-mutated drug targets (4, 12).

Reprogramming techniques cannot be considered as a new therapeutic approach per se. Since the pioneering findings of Huggins and Hodges in breast cancer, nuclear receptor (NR) agonists or antagonists have attained widespread use for therapy of hormone-sensitive tumors and hematologic neoplasias, and diversified NR ligands are still studied for extending indications, such as peroxisome-proliferator receptor α and γ (PPAR α/γ) agonists (13–17). Hormone-sensitive tumors comprise the most frequent tumors worldwide and are, importantly, not limited to sex-hormone dependent tumors (17). Based on these pivotal clinical results with NR ligands, and the increasing repertoire of drugs facilitating induction of differentiation, transdifferentiation, reconstitution of immunosurveillance and alternative tumor cell death, it is time to summarize these tumor tissue reprogramming approaches as a specific treatment paradigm to pave the way for systematic drug repurposing and vice versa to establish novel tumor systems states associated with clinically meaningful tumor control. Moreover, in a second step, edited tumor tissues provide novel biological access for targeted therapies, thus expanding the pharmacologic repertoire, for both, treatment of advanced cancer or hematologic neoplasias (4, 11, 18). The present review summarizes important examples of tumor tissue editing and targeting of edited tumor tissue to indicate the huge field of possibilities for systematically evaluating drug repurposing in metastatic tumor disease.

## Two-Step Drug Repurposing

**Figure 1:**
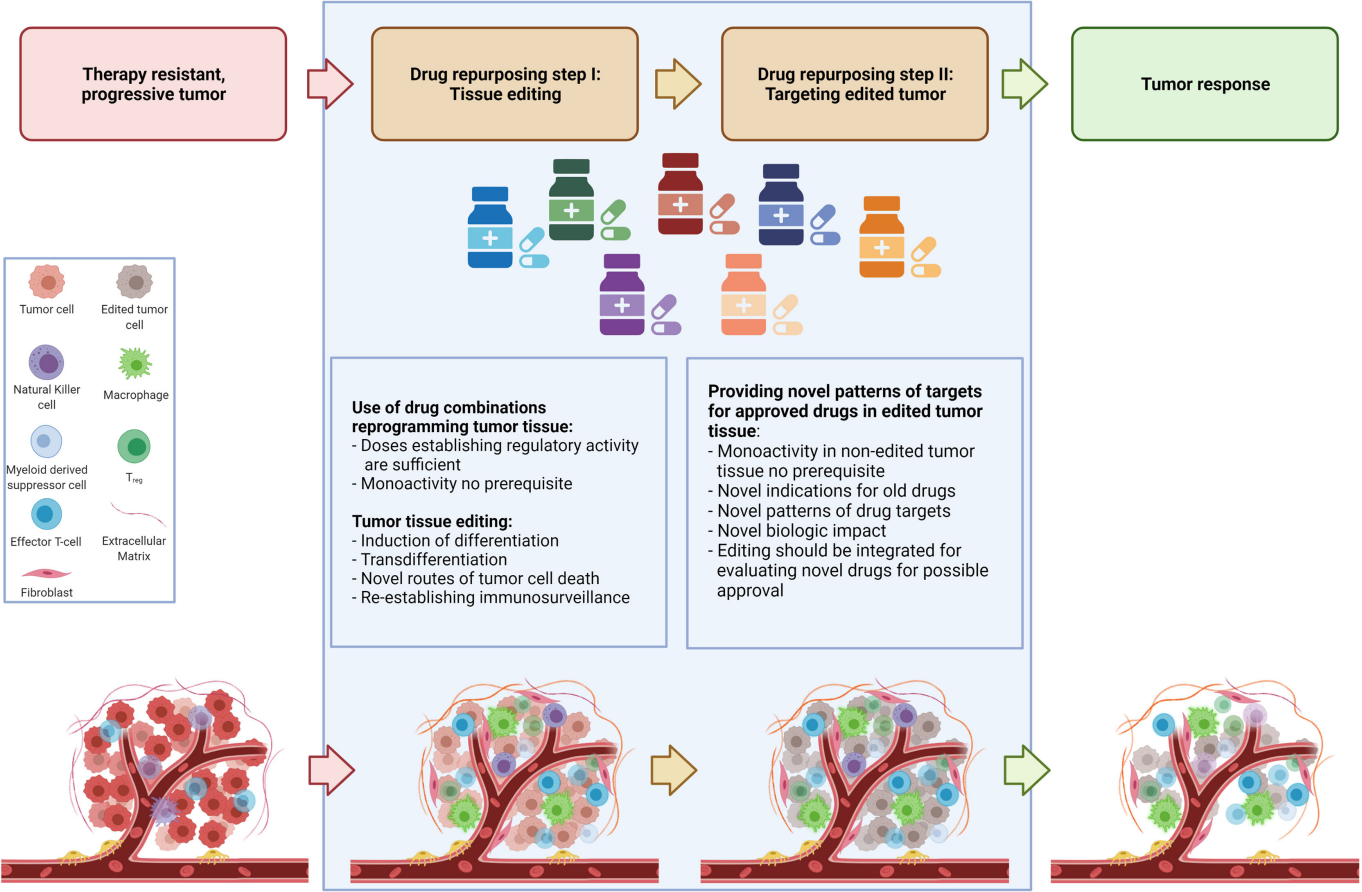
Establishing novel system states for tumor control in a two-step drug repurposing procedure.

For elaborating drug repurposing options in metastatic neoplasias, it is important to consider two separate identification processes for repurposing. The two-step identification of drugs for repurposing encompasses the selection of appropriate combinations of approved drugs with pro-anakoinotic activity profiles for establishing novel functional status for therapeutically ‘editing’ tumor tissues by induction of differentiation, transdifferentiation, or establishing immunosurveillance and tumor cell death etc. (5, 11, 19–23). The second step is to select approved targeted therapies for enhancing the biomodulatory effects or for establishing prerequisites for the induction of continuous complete remission (4, 6, 10, 24, 25). After tumor tissue editing, approved targeted therapies meet systems status on tumor sites which presumably have not been systematically evaluated pre-clinically and clinically. As shown, targeted therapies without or poor monoactivity can provide important impact on clinical outcome following tumor tissue editing, e.g. the addition of mammalian target of rapamycin (mTOR) inhibitors, azacitidine, α-interferon, cell cycle-dependent protein kinases four and six (CDK4/6) inhibitors, elotuzmumab etc. (9, 21, 22, 25–27).This separation in two steps is to some extent arbitrary, as both therapeutic steps are interwoven and strengthen one another as revealed by data on clinical trials (**Figure 1**).

The routine scheduling of classic targeted therapies commonly completely underestimates the diversity of tumor systems states and the important fact that activity profiles of single targeted drugs may context-dependently decisively change, as to be exemplified with mTor inhibitors (28–30). Tumor tissue editing provides the chance for reprogramming tumor systems functions towards biological hallmarks associated with tumor growth control and systematically expands a novel range of application for classic targeted therapies, either by targeting frequently overexpressed targets in tumor tissues, such as immune checkpoints, mitogen-activated protein kinase kinase MEK, mTor etc. or classic targets selected according to molecular-genetic aberrations in tumor cells (28, 31, 32). Following tumor tissue editing classic targeted drugs may be systematically used in a repurposed manner (**Figure 1**).

Two-step drug repurposing opens a window for establishing and evaluating novel tumor system states appropriate to establish biological tumor control. In some cases long-term control or even continuous complete remission or facilitating bridging to curative therapies, like allogeneic bone marrow transplantation may be achieved (9, 10, 25, 26, 33). Importantly, similar therapeutic reprogramming techniques can be used in histologic different tumor types (11).

## First Step Repurposing: Tumor Tissue Editing

### Designing Tumor Tissues’ Plasticity With Reprogramming Techniques

Cancer tissue’s plasticity is prerequisite for successful tissue editing. Plasticity may be considered as the ability of cell compartments in cancer tissues to change their phenotypes without additional genetic mutations in response to environmental requirements (34). Endogenous tumor tissue plasticity promotes varying tumor phenotypes to finally constitute hallmarks of cancer. Translational processes, metabolic reprogramming, and epigenetic mechanisms are main regulators of the tumor tissue’s plasticity (29, 35–37).

Single cell transcriptomics and genomic sequencing are steadily advancing our knowledge about the diversity of stable phenotypes promoted by one aberrant tumor genome (36, 38). Distinct phenotypes may even persist during several divisions, indicating that the phenotypic imprint represents a druggable target for cancer therapy and may be responsible for non-genetic tumor cell heterogeneity (39, 40). Besides the genetically driven spatial and topographic tissue, or cellular niche-associated heterogeneity, phenotypic heterogeneity arises, due to adaptive processes during tumor evolution (34).

The stability of cellular phenotypes in tumor tissues, and their diversity determines non-tumor cell autonomous tissue structures, functions, and hubs as ‘novel’ phenotypically derived therapeutic targets which seem to be much more diversified than genetic targets (12). The development of those, endogenously configured therapeutic targets, cannot always be directly linked to genetic aberrations. Their formation may rather be driven by communicative challenges in tumor tissues’ cellular compartments and niches.

The uncovered regulation of translational processes for establishing diversified tumor phenotypes and during several divisions persistent single cell transcription in tumor cells puts another complexion on the well-known epigenetic processes which are considered to establish stable phenotypes by biologic memory. In comparison to translational modification, epigenetic processes need the dynamics of the whole transcriptional machinery of a gene regulatory network, which is guided by ever changing strong transcriptional activators, the ‘memory’, guaranteeing the reversibility of systems states (37).

The amazing drug induced plasticity of cancer cells has been figured out by RNA sequencing analyses and uncovered plasticity of cancer cells in single cell spatial resolution (39, 40). Treatment failure is commonly interpreted as persistence of (residual) tumor cells but may be also ascribed to transcriptional modifications provoked by the presence of therapeutically altered tumor and stroma cells, besides the presence of apoptotic tumor cells, which probably serve as novel attractors for tissue remodeling or relapse (41). Thus, in any kind of response, therapy triggers far reaching phenotypic adaptions (42).

Therapeutically intended tumor tissue editing by systemic biomodulatory therapy with repurposed drugs, remains an often-unconsidered option by drug developers. By harnessing tumor cell and non-tumor cell plasticity the overall biological function of tumor tissue can be changed (40, 43) **Table 1**.

**Table 1:** Drug repurposing for tumor tissue editing; possible target tumors and substance classes for establishing novel biologic hallmarks facilitating attenuation or resolution of tumor growth.

Group of drugs used for editing tumor tissues(examples)	(Possible) indications	Drug characteristics	Possible editing results: novel tumor systems states
Nuclear receptor **agonists/antagonists**, transcriptional modulators	Solid tumors, hematologic neoplasias	• Combined regulatory activity • Monoactivity no prerequisite • Maximal tolerable doses no prerequisite • Regulatorily active doses of single drugs • Often metronomic scheduling • Often simultaneous targeting of tumor and stroma cells • Reprogramming communicative infrastructure of tumor tissue • Broad diversity of classic targeted therapies, but also ilow-dose chemotherapy	• **Induction of alternative pathways promoting itumor cell death** (Solid tumors, hematologic neoplasia) • **Induction of transdifferentiation of tumor cells** (i.e. in breast cancer) • **Restoration of tumor-immunosurveillance** (Solid tumors, hematologic neoplasia) • **Induction of differentiation** [Hematologic neoplasias (acute myelocytic leukemia) including acute promyelocytic leukemia]
(metronomic) low-dose chemotherapy	Solid tumors, hematologic neoplasias		
Fibrates	Lymphomas		
Anti-inflammatory drugs: coxibs (peroxisome-proliferator activated receptor α/γ (PPARα/γ) agonists, glucocorticoids)	Solid tumors, hematologic neoplasias		
Immunomodulatory imide drugs (IMiDs)	Multiple myeloma, lymphomas		
Vitamin D	Lymphomas		
miRNAs (currently not approved)	Solid tumors, hematologic neoplasias		

### Examples of Tissue Editing

#### Transdifferentiation

Transdifferentiation or endogenous epithelial-mesenchymal transition (EMT), are from a therapeutic viewpoint unfavorable evolutionary processes in tumor tissues, as novel, more aggressive tumor cell components often afford change of therapy concepts. Moreover, tumor transformation to a higher-grade neoplasia, as consequence of long-term systemic therapies, is frequently associated with additional molecular-genetic alterations (44–46).

Besides the possibility of a reciprocal switch between proliferation and differentiation, tumor tissues may induce EMT for establishing novel phenotypes. TGF-β modulates EMT-associated transcriptional networks. Phenotypic plasticity is one prerequisite for therapy resistance due to dedifferentiation, coupled with decisive environmental remodeling (47), e.g., by TGF-β induced fibrosis. Under physiological conditions, EMT facilitates migration of epithelial cells during tissue development, but also during wound healing. On a molecular basis, microRNAs care for homeostatic balance of the transcriptional network promoting EMT (48). During EMT in cancer tissue, RAS-responsive element binding protein 1 (RREB1) directly cooperates with TGF-β-activated SMAD transcription factors. The final manifestation of EMT is context-dependent due to varying chromatin accessibility of RREB1 and SMAD which facilitate activation of further transcription factors (49).

MEK, another downstream effector of TGF-β signaling, has been identified as gatekeeper for the promotion of EMT-induced cancer cell adipogenesis, as shown by repression of the MEK-ERK pathway (50, 51).. During EMT, TGF-β triggers two simultaneous effects, increased cancer cell plasticity *via* RREB1 and SMAD and inhibition of adipogenesis by the non-canonical MEK-ERK pathway (Su et al., 2020).

#### Differentiation

Cell differentiation is regulated by multiple genes and always a context-dependent multicellular process (52). The interplay of transcription and genomic conformation is driving cell-fate decisions (53, 54). The degree of differentiation in tumor tissues is related with malignancy and metastatic potential (55). Thus, the idea of differentiation induction in tumors is followed since the stimulating therapeutic success of all-*trans* retinoic acid (ATRA) and later arsenic trioxide in promyelocytic leukemia (APL) (20). Maturation arrest and the resulting proliferation have been thought to be irreversible (56). This working hypothesis is countered by therapy results in APL with differentiation inducing agents. Looked at more closely, however, the fact is that differentiation inducing agents, such as ATRA or arsenic trioxide, may exert quite differential additional anti-leukemia effects, besides differentiation (57).

Among these ‘classic’ differentiation inducing drugs, a series of newly approved classic targeted therapies may induce differentiation in non-APL leukemias. This newly arising therapeutic scenery casts light on the multifold mechanisms involved in differentiation processes and that re-establishing differentiation programs in proliferating neoplasias might afford differential therapeutic access for inducing clinically meaningful outcome (58).

Also in cancer tissues, complex non-tumor cell autonomous regulatory systems are essential to adaptively provide tumor promoting cellular phenotypes, a process which has been originally described for normal tissue by Waddington and is termed epigenotype (59).

Further, the translation system in tumors offers the possibility to set translational activity in two modes, proliferation, and differentiation. Stress response in hypoxia triggers the expression of a subset of *NANOG*, *SNAIL* and *NODAL* mRNA isoforms characterized by different 5’ Untranslated Regions (5’UTRs) promoting stem cell like features of tumor cells (29).

The influence of translational signatures on differentiation programs is therapeutically interesting, as not only mTOR inhibitors, but also cytotoxic drugs, induce cancer cell plasticity by enhancing translation of *NODAL, NANOG*, and *SNAIL* mRNA isoforms. Hypoxic tumor conditions are finally propagating accumulation of cancer stem cells and increase aggressiveness of cancer disease (29). Hypoxia and severe metabolic changes associated with differentially developing vascular supply represent differentiation opposing stimuli in tumor tissue (60).

Pharmacologic inhibition of these multi-level processes may prevent the transition to stem cell like tumor cell features. The experimental findings underline the non-genetic nature of an important tumor behavior, the reciprocal transition of mature and stem cell like states associated with respective phenotypical changes (29, 34).

In hematologic neoplasias, disruption of DNA methylation reshapes the hematopoietic differentiation scenery (61). A large series of drugs, e.g., histone deacetylase inhibitors (HDACi), histone methyl-transferase inhibitors (HMTi), and DNA metyl-transferase inhibitors (DNMTi) are suggested that their anti-tumor activity is at least partially mediated by targeting abnormal epigenetic patterns in tumor tissue inhibiting tumor cell differentiation. Just poorly differentiated tumor tissues show elevated activity of enhancer of zeste homolog 2 (EZH2), a histone-lysine N-methyltransferase enzyme, that is involved in histone methylation and consecutively, in transcriptional repression. HMTi blocks activity of EZH2 and may reinduce differentiation in neoplasia. Overall, cancer cell and tissue reprogramming with epigenetically active drugs seems to be a promising therapy, while facilitating the conversion of malignancy to a less aggressive tumor systems state (62, 63).

Azacytosine is a synthetic nucleic base and is chemically an analogue to cytosine and can be linked both to ribose and desoxyribose, resulting in the drugs Azacitidine and Decitabine respectively. The insertion of azacitidine in DNA and RNA inhibits DNA- and RNA-methyltransferases associated with a demethylation and may promote differentiation (64).

Inhibitor of differentiation 1 (ID1) is frequently overexpressed in glioblastoma cells, inhibits differentiation signals, and enhances MYC expression *via* activation of WNT and SHH signaling stemness. Thus, ID1 inhibition could re-establish differentiation in glioblastoma (65).

As heterodimeric cell surface receptors, integrins play an important role for proliferation and differentiation processes in tumors and impact therapeutic outcome (64). Differentiation is associated with morphological changes in cell and nuclear shape and mitochondrial distribution (66).

Acute myelocytic leukemias (AMLs) are eminently suitable to demonstrate therapy-related induction of differentiation both, morphologically and functionally (7, 67). Currently there is choice of a series of approved drugs, cytosine arabinoside (AraC), FLT3 inhibitors and inhibitors of the mutant IDH1/IDH2, all characterized to induce to some degree differentiation in AML. Another series of drugs, namely inhibitors of BET protein, DOTIL1 and HDAC, showed differentiation induction *in vitro*, and in animal models, but failed to show decisive clinical activity so far (67). To what extent tyrosine kinase inhibitors with differential activity profiles are active in AML patients without mutated FLT3 remains an open question addressed in ongoing trials. Their main anti-leukemia activity is probably not mediated *via* differentiation induction (68).

The discrepancy between preclinical results and the clinical benefit of targeted therapies with the capacity to induce differentiation hints that differentiation of AML blasts is a context-dependent process mediated by interaction with stromal cells and not exclusively *via* a leukemia cell-autonomous process (69).

In so far it is not surprising that just the combination of drugs, as already described, azacitidine and PPARγ agonists, modulating the whole communicative context in leukemia tissue combined with ATRA may induce hematologic complete remission in refractory AML. The often-short time range until hematologic remission demonstrates the therapeutic significance of non-leukemia cell autonomous targets in the bone marrow (25, 70–72).

The triple combination, low-dose azacitidine, ATRA and PPARα/γ agonist can induce differentiation as morphologically shown *in vitro* and *in vivo*. Moreover, it also re-establishes phagocytic activity of these differentiated blasts. This is underlined by findings in patients who resolved fungal pneumonia while being treatedwith this combination. Therefore these morphologic changes are additionally associated with a gain of function which can be seen experimentally and clinically (25).

#### Re-Establishing Immunosurveillance

Successful immune checkpoint inhibitor (ICI) therapy strongly depends on the immune environmental context, as expression of immune checkpoints is widely distributed among stromal and tumor cells. Additionally, immune cells may be differentially compromised by the tumor tissue, thus impeding clinically meaningful immune response with ICIs (73, 74). Important therapeutic approaches for re-establishing immunosurveillance comprise adaptive regulation of chemokines and cytokines contributing to immune escape, metabolic reprogramming of T cell function, up-regulation of tumor suppressor genes, e.g., Phosphatase And Tensin Homolog (PTEN), enhancing antigenicity of cancer cells (73, 75–80).

#### Re-Establishing Pathways Inducing Tumor Cell Death

Both, pre-clinical and clinical data reveal that anti-estrogen therapies reduce tumor cell proliferation, arrest tumor cells in the G1 phase of cell cycle (81). These are all processes which may finally re-establish induction of apoptosis (82). However, re-establishing apoptosis in breast cancer cells may be only one aspect of estrogen receptor antagonism considering the thousands of estrogen receptor target genes, many of them regulating proliferation (83). Editing breast cancer tissue with anti-estrogen therapies provides the possibility to enhance pro-apoptotic processes by blocking the frequently occurring up-regulation of cyclin D1 in breast cancer cells with CDK4/6 inhibitors (84). In this way CDK4/6 inhibitors gain activity which translates in significantly improved progression-free survival in contrast to modest monoactivity of CDK4/6 inhibitors, even in endocrine resistant breast cancer (10).

Restoring or side-lining the functional impact of recurrently occurring mutations, such as mutations of the tumor suppressor TP53 by re-establishing differentiation and tumor cell death is of great therapeutic interest. Accumulation of α-ketoglutarate reestablishes p53 function in cancer cells. By enhancing α-ketoglutarate levels in p53 deficient tumors, tumor cell differentiation may be initiated (85) Arsenic trioxide may re-establish the apoptotic capability by mutant p53 and therefore, could be used for rescuing the tumor suppressor function in a broad variety of tumors (86).

Fibrates may induce apoptosis in lymphomas by inhibiting the TNF alpha/NF-kappaB signaling axis. As fibrates are PPARα agonists they edit the whole lymphoma tissue as PPARα is expressed on lymphoma and stroma cells (87).

#### Exploiting Metabolic Vulnerabilities

A growing tumor needs its metabolism to fuel a constant demand for new building blocks and energy in order to survive. This gives rise to new vulnerabilities that can be exploited in tumor therapy. Several well characterized cytostatic drugs are essentially interfering with tumor metabolism. I.e. 5-Fluorouracil is inhibiting thymidine synthesis and Methotrexate interferes with the tetrahydrofolate metabolism, limiting supply of one carbon building blocks i.e. for amino acid biosynthesis. There are several other medications like metformin or hydroxychloroquine that are approved for different treating diseases that may be repurposed in an pro-anakoinotic fashion (88, 89). Metformin is an inhibitor of complex I in oxidative phosphorylation and also influences the PI3K/AKT mTOR pathway, which are both interesting targets in cancer therapy. Additionally Metformin appears to have an impact on neoangiogenesis (90). Especially in gynecologic malignancies there is, mostly observational, evidence that diabetic women treated with metformin might have a benefit from this therapy. Unfortunately there is so far no conclusive evidence from large scale clinical trials, that women with gynecological cancers benefit from additional metformin treatment (91). This might be due to the retrospective nature and heterogeneous study populations of the trials published so far. Clinical trials exploring this question are under way (91). Because of its pleiotropic effects, we believe there is great potential for metformin to be incorporated into a biomodulatory repurposed approach.

#### Re-Thinking Drug Formulation

Many drugs have shown to be effective *in vitro* but failed to show their efficacy *in vivo*. Effectively delivering a drug to its intended location of action is an often overlooked hurdle that needs to be overcome in drug development. Especially lipophilic drugs are usually limited by their low solubility in aqueous conditions. One example are retinoids. These aliphatic derivatives of vitamin A have a strong impact on cellular metabolism as demonstrated by ATRA’s impact on treatment of acute promyelocytic leukemia (20). Another example of this class of drugs is fenretinide. While showing promising activity *in vitro*, clinical trials failed to show efficacy, partly due to low bioavailability (92, 93). By using nanoparticle formulations their bioavailability can be increased to a point where they can fully exert their biologic potential (92, 94). One more example for enhancing efficacy by re-formulation is CPX351. This stochiometrically fixed and nanoparticle packaged combination of cytarabin and daunorubicin has had a favorable impact on AML therapy, improving overall survival when compared to conventional routes of application (95). In conclusion drug repurposing should also take alternative drug delivery systems into account as they might contribute significantly to tumor tissue editing effects.

### Tumor Tissue Editing: Providing Novel Targets

Tumor tissue editing may establish access to completely novel phenotypic contexts in tumor tissues to promote either differentiation, transdifferentiation or re-establish immunosurveillance and apoptotic capabilities.

#### Overcoming Therapeutic Bottlenecks

Differentiation or transdifferentiation may bring along neoantigens or altered antigenicity (96). Enhancing tumor antigen frequency could be a pivotal step induced by tumor tissue editing. On the background that ICIs are highly efficacious in a broad variety of cancers and that a broad diversity of tumor-specific lymphocytes is necessary to enhance ICI activity profiles, tumor tissue editing seems to be of pivotal interest to improve clinical efficacy of ICIs (8). Such a therapeutic procedure could be also of advantage for the use of neoantigen-directed T-cell-receptor (TCR)-engineered cells (97). A further aspect of tumor tissue editing is that the immunosuppressive microenvironment may be reconfigured to abandon T-cell suppression for improving immunotherapy.

For example, abnormal Wnt/β-catenin signaling is frequently found in tumor tissues, thereby, functionally involving the whole immune compartment (98). Pro-anakoinotic therapy with PPARγ agonists may attenuate Wnt signaling as both pathways can be regarded antagonists, thereby concertedly inducing differentiation, enhancing immune response, reducing tumor-associated inflammation, angiogenesis and cell proliferation (99, 100).

Each treatment procedure leaves phenotypically characteristic biologic memory in tumor tissue and/or novel acquired (epi)genetic signatures in tumor cells (101). These evolutionary processes determine drug sensitivity during further tumor progression or relapse. Protein-coding genetic aberrations frequently do not sufficiently explain arising drug resistance (102).

Genetic heterogeneity developed during tumor evolution/preceding systemic tumor therapy provides a principal obstacle for up-coming systemic therapies and may frequently be the cause of mixed response. In such therapeutically complex system states, non-cancer cell autonomous and thereby constant structures and functions provide a novel pattern of clinically important targets for tumor control and contribute to address a novel understanding of ‘treatment resistance’.

Tumor cell niches at primary and metastatic tumor sites are up-to-now not in focus of systemic tumor therapy. Nevertheless, in these circumscript topographic niches heterotypic cell types either contribute to tumor growth or keep tumor outgrowth at bay. It seems to be a domain of pro-anakoinotic therapy to moderate the communicative network in niches for attenuating or inhibiting tumor growth (103). Clinical evidence that tissue editing can target metastatic niches is derived from phase II trials in patients with advanced malignancies. In about 60% of the cases clinical relapses of metastatic refractory disease following pro-anakoinotic therapy occur at initial tumor sites and not at novel sites (104). Thus, targeting non-tumor cell autonomous targets seems to be efficacious in controlling novel metastatic outgrowth. Therapeutic control of the metastatic niche could impact cancer cell stemness and consecutive invasiveness of tumor cells (105).

#### Transcriptional Addiction

Developing non-tumor cell autonomous structures, and functions enable tumor tissues to configure a distinct malignant phenotype as diagnosed by highly specific histologic tumor types (106). Mutations in cancer cells re-integrate environmental signals by highly specific communication-driven transcriptional responses resulting in phenotypic changes finally altering cell fate in comparison to the originally normal cellular or tissue counterpart. Environmental attractors are alternatively interpreted by the tumor cell leading to the activation of aberrant transcriptional networks which lead to a stabilized regulatory network supporting tumor growth. Tumor type specific super-enhancers play a decisive role in this adaptive process and could serve as specific therapeutic targets (107, 108). If oncogene addiction by a driver mutation is highly developed, direct therapeutic inhibition of super-enhancers seems to be promising (109).

The novel transcriptional systems state in tumor tissues may be targeted by tumor tissue editing *via* transcriptional modulators. Differentiation therapy with ATRA for acute promyelocytic leukemia (APL) serves as a pivotal example for targeting transcriptional addiction due to the retinoic acid receptor alpha (*RARA*) fusion with a second gene (*PML)* specifying a novel Krüppel-like zinc finger protein, but also as an example explaining leukemia tissue editing by differentiation induction (20, 110). While considering differentiation phenomena in APL, the transcriptional addiction in APL is frequently overseen. However, this is of great importance if the differentiation approach shall be operatively transferred to other neoplasias.

While differentiation programs are still available to be initiated by pro-anakoinotic therapies in many kinds of cancer tissue, they specific to a certain tissue. Access to these programs depends on both, the acquired oncogenic drivers and the multifold evolving non-tumor cell autonomous structures and functions (i.e. target organ, T-Cell composition, extracellular matrix, etc.) in tumor tissues specifying a specific tissue phenotype.

An early example of clinically well-established reprogramming is antihormonal therapy in prostate and breast cancer. In these tumors androgen and estrogen receptors are commonly not mutated and their ligands can exert their function to maintain tumor growth (111–113).

As transcription is a dynamic networking system, functions and hubs may be therapeutically edited *via* transcriptional modulation to exploit pivotal bottlenecks, as for example, available in hormone receptor positive breast and prostate cancer (17). Nuclear receptors facilitate differentiation and regulate time-sensitive growth during evolutionary states (17, 114, 115). Importantly, potential vulnerabilities of communication protocols regulating transcriptional networks are not necessarily predictable by acquired genetic changes (106).

#### PPARγ Agonists and Tumor Tissue Editing

PPARα/γ are ubiquitously distributed among normal and cancer tissues in a tissue specific manner, thus immediately depicting an important role in cell-cell communication (100). Targeting PPARα/γ with corresponding agonists (i.e. Pioglitazone or Rosiglitazone) means that context dependent responses are challenged which may not be easily predicted by *in vitro* assays. The biologic read outs finally represent the concerted diversified activity profiles which perfectly meet the idea of tumor tissue editing, differentiation induction and transdifferentiation. Normal tissue of the tumor hosting organ as well as tumor tissues may be edited in parallel, importantly in a clinically meaningful way (100).

The multifaceted activity profile of PPARα/γ agonists in tumor tissue may be best exemplified in leukemia, both, in chronic myelocytic leukemias in combination with imatinib and in acute leukemias in combination with ATRA and low dose azacitidine (7, 25, 103, 116, 117). In parallel, normal hematopoiesis may be supported in contrast to the expected side effects, namely long-term suppression of hematopoiesis, following chemotherapy or many other targeted therapies, such as for AML therapy approved bcl-2 inhibitors (7, 25, 71, 118).

According to the diversified editing activities, PPARα/γ agonists can be combined with all kinds of systemic therapies, namely pulsed chemotherapies, metronomic chemotherapies, targeted therapies, radiotherapy, immunotherapy and anti-inflammatory therapies (11, 119). Additionally, novel therapeutic qualities may be achieved by two-step drug repurposing according the novel treatment model (6, 11, 116).

## Second Step Repurposing: Matching Drug and Tumor Diseases’ Systems State

Drug-disease matching generally applies to distinct histologically or molecular-genetically described tumor types. Therapeutically underappreciated is matching of targeted therapies with distinct functional states of tumor disease. Therapeutic attempts to ‘synchronize’ the functional status of tumor tissues caused by reprogramming approaches are common in endocrine therapies, but otherwise rarely used in oncologic praxis (**Table 2**) (20, 33).

**Table 2:** Drug repurposing in edited tumor tissue; possible matching drug and tumor diseases’ systems state by drug repurposing.

Drugs administered inedited tumor tissue (examples)	(Possible) indications	Drug characteristics/Edited drug targets	Clinical results
mTOR inhibitors	Hodgkin’s disease, melanoma, breast cancer	Classic targeted therapies Tumor cell autonomous and non-tumor cell autonomous targets Maximal tolerable doses no prerequisite Combined regulatory activity Novel patterns of drug targets Novel biologic impact of ‘old’ but also experimental drugs	Control of refractory tumor disease or hematologic neoplasias Remission Continuous complete remission Long-term disease control Improvement of progression-free survival Improvement of survival Beneficial progression-free survival 2 Bridging for allogeneic blood stem cell transplantation Chemoprevention
MEK inhibitors	Melanoma, Breast cancer		
KRAS inhibitors	Non-small cell lung cancer, gastrointestinal tumors		
Elotuzumab, proteasome inhibitors	Multiple myeloma		
Monoclonal/bispecific antibodies against tumor cell epitopes CART-cells	Hematologic neoplasias (solid tumors)		
CDK4/6 inhibitors	Breast cancer (prostate cancer)?		
α-interferon	Renal cell carcinoma, Langerhans cell histiocytosis		
Immunotherapies: checkpoint inhibitors, vaccines, cytokines, chemokines, toll-like receptor agonists	Multiple types of cancer and hematologic neoplasias		
Epigenetic modifiers	Myelodysplastic syndrome, acute myelocytic leukemia		

The matching of drug and tumor diseases’ systems state comes into focus with the introduction of the anakoinosis paradigm aiming at establishing a novel functional systems status in tumor tissues. On basis of multifold clinical data, it turned out that anakoinosis is a generally applicable therapeutic principle in quite different histologic tumor types and that the anakoinosis paradigm is already followed in established treatment schedules, for example in endocrine combination therapies or in many therapies for multiple myeloma (10, 24). Therefore, novel attempts for matching efficacy of specific targeted therapies on basis of novel therapeutically edited starting positions, namely the novel arranged distribution of drug targets arise as approaches which can be universally applied to identify drugs for repurposing.

Identifying drugs suitable for repurposing, cannot leave out of consideration that tumor tissues always represent a characteristic imprint of functional, communicatively derived systems status, which are therapeutically accessible *via* biomodulatory approaches (11). Endogenous plasticity is characterized by the possibility of tumor tissues to apply multiple non-tumor cell autonomous processes for modulating tumor phenotype, namely epithelial-mesenchymal and mesenchymal-epithelial transition and the switch from more differentiated tumor cell phenotypes to stem cell like ones and vice versa (35). Moreover, EMT is strongly affected by the communicative infrastructure in the tumor tissue that means also from non-tumor cell autonomous functions in the tumor microenvironment (120, 121). The addition of classic targeted repurposed therapies, commonly with insufficient monoactivity in the respective tumor entity, may contribute to continuous complete remissions in edited refractory tumor disease (11, 33, 122).

Anakoinosis has been clinically studied in a broad range of histologically quite different neoplasias, yet insufficiently pre-clinically. A limited number of pre-clinical data exemplifies, that differentiation induction, or successful, therapeutically relevant induction of epithelial-mesenchymal transition may provide significant tumor response, clinically or in animal models (6, 7, 25, 123, 124).

Pro-anakoinotic therapies open a huge field for repurposing drugs, just for treatment of refractory tumor diseases. Therefore, the identification process of drugs suitable for repurposing should be systematically enhanced and promoted. Anakoinosis inducing therapies may provide novel repurposed indications for ‘old’ targeted therapies. Potentially developmental drugs that have not reached phase I clinical trials or clinical approval because of lack of efficacy in their primarily intended indication could also be retested in anakoinotic setting. Therapeutically inducible, differential functional systems status in tumor diseases have not been in the focus of pre-clinical evaluation, yet. Beyond drug activity in distinct histologic tumor types, specific therapy for edited tumor tissues, may be important, as the functional status of edited tumor systems might be shared by histologically different tumor types. Indeed, quite different tumor types may be treated with similar pro-anakoinotic treatment schedules (4, 11).

In the following section, targeted drug activities are discussed on basis of parallel or preceding tumor tissue editing in an exemplary way.

### CDK4/6 Inhibitors and Tissue Editing With Endocrine Therapy

CDK4/6 inhibitors in combination with endocrine therapies, e.g. tamoxifen, aromatase inhibitors, fulvestrant, in ER-positive breast cancer seem to be a ‘prototypic’ example for transcriptional tissue editing combined with repurposing of targeted therapy as indicated by enhanced combinatorial efficacy of CDK4/6 inhibitors compared to monotherapy (10, 27, 125). CDK4/6 inhibitors have been approved only in few clinical indications, although, considering the activity profile CDK4/6 inhibitors, they should work in many histologic tumor types (126, 127). The point is, CDK4/6 inhibitors start to improve progression free survival significantly when supported by biomodulatory endocrine therapy, for example in breast cancer. Prostate cancer trials are on the way with enzalutamide (128). Endocrine therapy contributes to multifaceted tissue editing, not only in case of classic drugs, such as tamoxifen, aromatase inhibitors, fulvestrant. Also, on experimental basis, nuclear receptor agonists, like PPARα/γ agonists combined with all-trans retinoic acid unfold activity by modeling stroma and tumor cells, thereby reducing aromatase activity in neighboring fat cells in the breast and negatively impact breast cancer stem cell survival (129, 130). Thus, tumor tissue editing provides the basis of repurposed CDK4/6 inhibitors associated with strong clinical activity. Alternatively, multifold trials are on the way combining CDK4/6 inhibitors with additional targeted therapies, such as mTor inhibitors, ICI, ibrutinib, bevacizumab etc. (127) for improving the modest palliative data of CDK4/6 inhibitors in monotherapy.

### KRAS Inhibitors and Tissue Editing With PPARγ Agonists

Kirsten rat sarcoma virus (KRAS) is a frequently mutated oncoprotein, characterized by a gain of function in tumor cells in a broad variety of histologically different tumor types. GD12D, is the most prevalent and meanwhile, targetable mutation (131, 132). The long history of failing approaches to target the ‘undruggable’ oncogene is now crowned with success with the first approved inhibitors of the KRAS p.G12C mutation. Best responses were observed in heavily pretreated patients with NSCLC, in about one third, but only in 7% of gastrointestinal tumors (133). The functional activity of mutant KRAS in mice models is dependent on activation of the nuclear receptor PPARγ. PPARγ-deficiency promotes pre-malignant phenotypes, initiated by mutant KRAS (134). Important is the multifaceted activity profile of PPARγ agonists in tumor tissues, characterized by antagonizing tumor-associated inflammation, promoting immune response and differentiation of tumor cells (135). Yet, no clinical trials have proven the combinatory use of KRAS inhibitors with parallel tumor tissue editing by PPARγ agonists. However, experimental data support the combination (134). The parallel use might repurpose the highly variable, often poor activity profile of KRAS inhibitors depending on tumor histology. Again, basis for repurposing could be edited tumor tissue. The double inhibition of the mutant driver oncogene, on tissue level with PPARγ agonists, reprogramming inflammation, immune response, inducing tumor cell differentiation and on tumor cell level with KRAS inhibitors, might attenuate the proliferative stimulus, and could be in future a suitable preventive and therapeutic approach (99, 134).

### mTOR Inhibitors and Tumor Tissue Editing

mTOR signaling is activated in many neoplasias and therefore, lends itself as promising therapeutic target (136). Except for renal cell carcinoma and mantle cell lymphoma monotherapies with mTOR inhibitors achieve poor clinical results (137, 138). A main reason for the modest efficacy of mTOR inhibitors may be the pleiotropic activity profile of mTOR characterized by interaction with multiple hallmarks of cancer, such as proliferation, tumor metabolism, and immune response, besides autophagy (28). All hallmarks are context-dependently constituted by the tumor tissue and as such it is not surprising that pre-clinical data on mTOR inhibitors are contradictory (28, 139). At the cutting edge between differentiation and proliferation control, mTOR inhibitors promote in the experimental setting translation, particularly of frequently available oncoproteins, like cMyc, cyclinD1, ornithine decarboxylase (ODC), vascular endothelial growth factor (VEGF), fibroblast growth factors (FGF), hypoxia-inducible Factor 1α (HIF1α) and myeloid cell leukemia sequence 1 (Mcl-1) and shift cancer cell phenotypes to cancer stem cell characteristics (29).

Not only the controversial *in vivo* and *in vitro* results with mTOR inhibitors demonstrate the difficulties to predict outcome to mTor inhibitors, but also the heterogeneously altered components of the mTOR pathway in tumor cells impact functionality of mTOR signaling, starting with the frequently reduced levels/mutations of the tumor suppressor gene *PTE*N or mutations/amplifications of *Phosphoinositide 3-kinase* (*PI3K*). All pathway components have important impact on tumor progression (140).

Due to the difficult predictability of response to mTOR inhibitors and missing general predictors, tumor tissue editing could provide a prerequisite to enhance efficacy of mTOR inhibitors (141). For example, mTOR inhibitors enhance immune checkpoint expression. The combination with ICIs is at least promising in pre-clinical trials., as immunosurveillance may be reestablished by increase of tumor infiltrating T-cells and a parallel decrease of regulatory T-cells (142, 143). Other possible combination partners could be CDK4/6 inhibitors or PI3K inhibitors (144).

Clinically, tumor tissue editing can adequately design tumor tissues, a prerequisite for efficacious use of mTOR inhibitors in refractory metastatic uveal melanoma or refractory Hodgkin’s disease with metronomic low-dose chemotherapy and a PPARα/γ agonist (21, 23). In uveal melanoma long-term disease stabilization with significant improvement of ECOG status was achieved, in refractory Hodgkin’s disease even induction of complete remission, moreover continuous complete remission after discontinuation of the metronomic schedule (26). In both tumor entities, monotherapy with mTOR inhibitors has modest, if at all any activity. However, tumor tissue editing in refractory systems states prompted a novel therapy quality of mTOR inhibitors, here everolimus or temsirolimus, respectively, which could not be achieved with the tissue reprogramming therapy alone (unpublished data). These preliminary clinical results represent pivotal examples for drug repurposing by tumor tissue editing (21, 26). In breast cancer, everolimus may significantly prolong progression-free survival of exemestane monotherapy in a phase III trial (145).

### α-Interferon Therapy Following Tissue Editing

Tissue editing in refractory, metastatic renal clear cell carcinoma (mRCC) with metronomic low-dose chemotherapy and pioglitazone, a PPARα/γ agonist, lead to disease stabilization. The addition of an approved drug in mRCC, α-interferon, now administered at very low doses weekly, in contrast to the approved and commonly used high doses in the era before introduction of targeted therapies, facilitated induction of histologically confirmed continuous complete remission in these advanced stages (22, 33, 146). The strong anti-inflammatory response mediated by α-interferon was significantly associated with response (147). In the setting of refractory, metastatic RCC, repurposing of α-interferon, now at very low doses, on the background of parallel tumor tissue editing could initiate long-term response in metastatic, refractory mRCC. In contrast, the addition of approved high dose α-interferon to temsirolimus, an mTOR inhibitor, or bevacizumab did not improve survival (148, 149).

### Immune Modulating Cytokines and Tumor Tissue Editing

Epigenetically based tumor tissue modeling may lead to a down-regulation of the frequently overexpressed oncogene cMyc, thus recovering interferon response, and down-regulating multiple chemokines (73, 78). Currently a number of approved immune stimulating cytokines are in evaluation, such as, IFN-α, IFN-β, IL-2, and GM-GSF, others are in first clinical trials IL-12, IL-15, and IL-21 (77). Immune-modulatory and anti-inflammatory treatment approaches, as well as metabolically reprogramming therapies are currently tested (11, 100, 150–155).

Summarizing the possible approaches to enhance immunosurveillance by reprogramming tissue functions, there seems to be huge therapeutic potential for repurposing of approved drugs in the intention to clinically improve efficacy of ICI therapy or even to circumvent resistance. The anakoinotic approach may contribute to a successful repositioning of ICI therapies in case of ICI resistance and progressive disease (8, 156).

### MEK Inhibitor Therapy Following Tumor Tissue Editing

Among the downstream kinases MEK plays therapeutically an important role, besides, mTOR, BRAF, AKT, as MEK is frequently up regulated in tumor cells. MEK inhibitors (MEKi) are approved in BRAF mutated melanoma and NSCLC in combination with BRAF inhibitors (157). Studies of MEKi in combination with CDK4/6 inhibitors are on the way in a broad variety of metastatic cancers (158).

The selective inhibition of the noncanonical TGF-β pathway involving MEK-ERK signaling, paralleled by the promotion of adipogenesis and inhibition of tumor-associated inflammation *via* PPARγ agonists may irreversibly differentiate breast cancer cell into adipocytes (6, 100, 159). Clinical trials indicate that PPARγ agonists may inhibit metastatic spread (104), what could be confirmed in the mouse model by treatment of metastatic breast cancer with rosiglitazone and MEK inhibitor (6).

The combinatorial use of MEKis with additional inhibitors of non-cancer cell or cancer cell autonomous signaling pathways follows a currently classic therapeutic procedure (160).

Simultaneous PPARγ activation and MEK inhibition combines the two therapy steps, tissue editing and targeted therapy in the edited tumor tissue. Tumor tissue editing is possible with PPARγ agonists. The stimulatory activity of PPARγ agonists changes phenotypes and functions of both, malignant and non-malignant cells and their respective ‘communication protocol’ due to the ubiquitous, but differential expression of PPARγ in all cell compartments of the tumor tissue (100). Following therapeutic editing of tumor tissue with PPARγ agonists, inflammation control and enhanced immunosurveillance may be achieved (11). The parallel abandoning of non-canonical TGF-β activity with MEK inhibition positions the function of the MEK inhibitor in a novel context. Both, MEK inhibitors and PPARγ agonists are used in a novel function compared to the common ways of application, namely for efficacious targeted control of *BRAF* mutated metastatic melanoma in combination with *BRAF* inhibitors or for overcoming insulin resistance, respectively.

### Epigenetic Modifiers Following Tumor Tissue Editing

Most trials with epigenetically modifying drugs have been successfully performed in hematologic malignancies, particularly myelodysplastic syndromes and acute myelocytic leukemia. The group of epigenetically active drugs comprises quite different agents, DNA hypomethylating, i.e., 5-azacitidine and 5-aza-2-deoxy-cytidine (decitabine), histone deacetylase inhibitors (HDAC inhibitors), and agents targeting the polycomb repressive complex (PRC) 2 factor EZH2, or BET protein family epigenetic readers, affecting transcription of genes with super-enhancers (BET inhibitors).

The reprogramming character of azacitidine, for example, can be revealed from response data in p53 positive higher-risk myelodysplastic syndromes (MDS) or secondary acute myeloid leukemia (sAML). Interestingly, response is independent of the poor risk prognostic marker, mutated p53, in MDS/sAML (161). The classic and approved combinatorial use of azacitidine is currently the combination with the bcl-2 inhibitor venetoclax (162).

The resumption of the reprogramming approach with azacitidine has been initiated in clinical trials adding differentiation inducing agents, such as pioglitazone and all-trans retinoic acid to the epigenetically modifying approach. Rapid hematologic complete remission may be achieved with the triple combination even in p53 positive acute myelocytic leukemia. Interestingly, phagocytic activity of differentiated AML blast may be restored and may lead to regression of severe fungal pneumonia during rescue therapy with the triple combination (7, 25, 70, 72).

### Propranolol and Tumor Tissue Editing

Pre-clinical and clinical evidence reveals that β-2 adrenergic receptor (ADRB2) signaling contributes to the progression of neoplasias, particularly in vascular sarcomas, prostate and breast cancer and may contribute to resistance (163–165). There is consistent evidence for at least additive activity of β-2 adrenergic receptor (ADRB2) blockers with chemotherapy (166).

Both, castration-resistant prostate cancer, and vascular tumors have been shown to be therapeutically accessible with reprogramming, pro-anakoinotic therapy approaches (167–169). Thus, the repurposed use of β-2 adrenergic receptor plus a tissue editing reprogramming approach could be possibly introduced for control of refractory prostate cancer and vascular tumors (170).

### Imatinib and Tumor Tissue Editing

The effect of PPARγ agonists to overcome imatinib resistance has been described as ‘purging’ or sensitizing of imatinib (171). Target of PPARγ agonists in CML are quiescent stem cells, thereby decreasing transcription of STAT5 (103).The combination PPARγ agonists plus imatinib has been tested in cases of insufficient molecular response in chronic myeloid leukemia (CML), although PPARγ agonists have no monoactivity in CML, and imatinib had no sufficient monoactivity. The combined activity may induce CML cell death and durable molecular response even after therapy discontinuation (116).

### Targeted Therapies and Tumor Tissue Editing, e.g., in Multiple Myeloma

Meanwhile, lenalidomide plus dexamethasone may be considered as a basic, clinically relevant, biomodulatory therapy active in all treatment phases of multiple myeloma, including maintenance or consolidation therapy (172). Lenalidomide’s mode of action can be considered as biomodulatory as it, in addition to its direct cytotoxic effects, inhibits angiogenesis, stimulates T-cells and natural killer cells and reduces inflammation (173). Additionally, both drugs combined show more than additive clinical activity in comparison to single agent activity (172). Clinical outcome may be improved by the addition of other biomodulators, such as proteasome inhibitors or monoclonal antibodies, like daratumumab, and elotuzumab (9, 24, 174–177). Adding pembrolizumab, however, seems not to be beneficial (178). The ‘editing therapy’, lenalidomide plus/minus dexamethasone may even have positive impact on progression-free survival 2 (179).

## Special Prerequisites for Reprogramming Drugs

The reprogramming technique imposes some special requirements on drug activity. Drugs, simultaneously active in tumor and stoma cells may be used, such as metronomic chemotherapy and NR agonists/antagonists. Combined regulatory activity, not necessarily monoactivity in tumor tissues is required, as exemplified for mTOR inhibitors, α-interferon, chemotherapy, PPARα/γ agonists. Not only antagonistic, blocking activity but also agonistic activity, e.g. NR agonists can be used (9, 26, 100, 180). For establishing reprogramming activity profiles of repurposed drugs, even lower doses may be sufficient, in contrast to the maximal tolerable doses required for classic targeted therapies to ensure monoactivity, as exemplified for azacitidine, α-interferon, and chemotherapy (4, 25). As shown in clinical trials, pro-anakoinotic therapies are even successful in refractory, metastatic neoplasias of highly different histologic origin beyond the classic sex-hormone related cancers (4).

## Experimental Approaches and Future Perspectives

**Figure 2:**
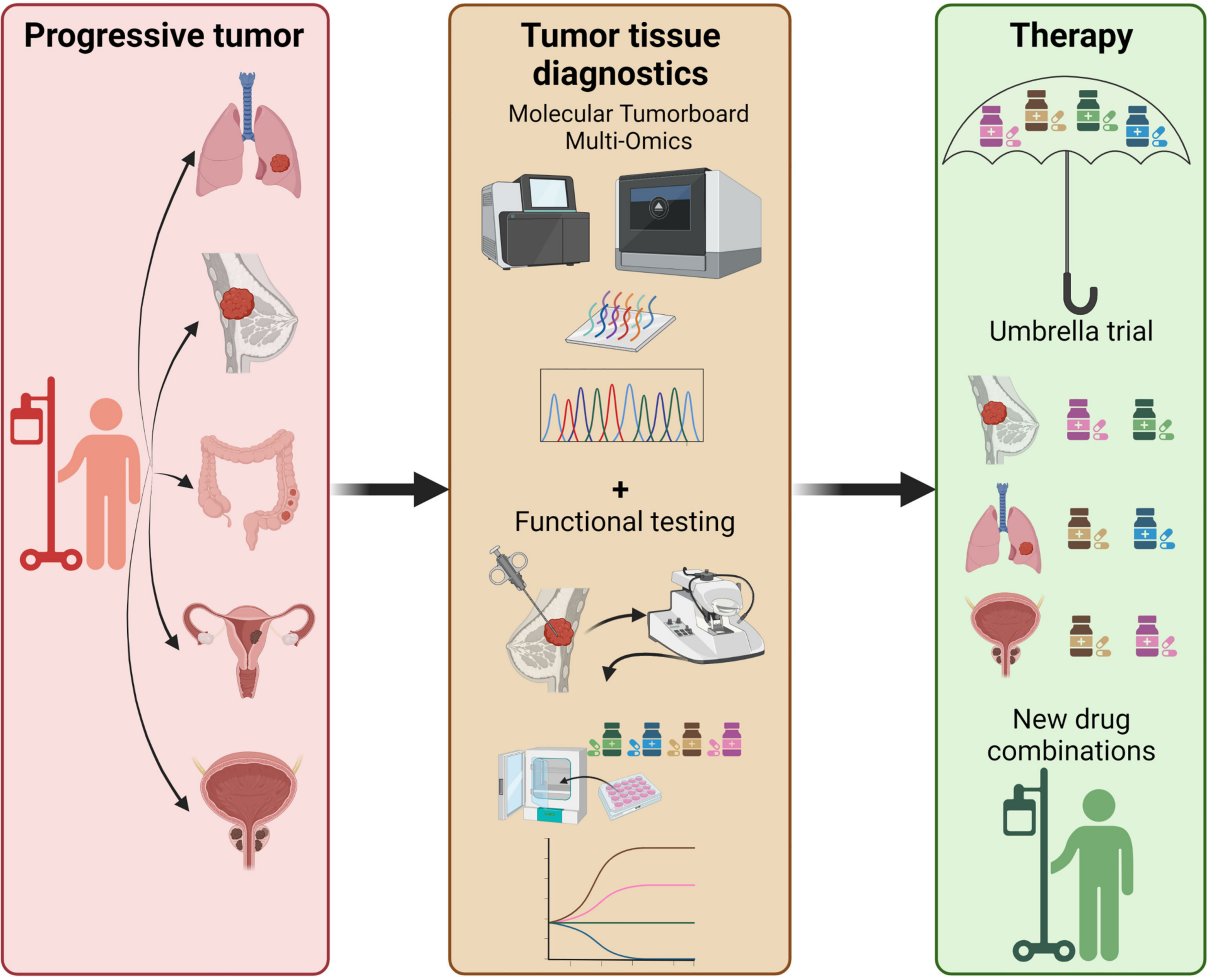
Perspective on future precision oncology platforms integrating molecular + functional data to predictively select the optimal drug combination, i.e. within an umbrella trial. Functional testing includes, but is not limited to, *in vitro* drug screening on organoids, precision cut tissue slices, tumor fragments; addition of autologous T-cells may also be considered to improve precision medicine approaches in an individual patient.

For clinical data re-interpretation on pro-anakoinotic treatments, however, explanatory experimental and computational derived data are only to some extent available. Such technological efforts may include pathway or signature matching, genome-wide association studies, computational molecular docking, evaluation of drug-disease and drug-drug similarities, imaging mass cytometry (IMC), and ‘signature’ matching in comparison to other drugs with proteomics, transcriptomics and metabolomics (1, 4, 7, 181–184). Moreover, phenotypic screening of tumor models comprises a very valuable tool for drug repurposing (1). Looking at tumor tissue editing it is even more challenging to develop a suitable screening/diagnostic platform, since the effects not only on tumor cells, but on all cells and structures in the TME and their interactions have to be considered. Classic approaches for drug repurposing using established tumor cell lines and humanized mouse models lack multiple, possibly critical components of the original tumor tissue. *Ex vivo* precision-cut tissue slices derived from patients’ tumors represent a promising approach to shed more light on tumor tissue editing (step 1) in the future (185–187). By using this technique the effects of various compounds and their combinations on heterogeneous tumor cells in their native tumor immune microenvironment could be investigated (188, 189). Protocols to embed precision cut tissue slices into Omics analyses have also been developed (190). This unique setting even gives rise to the possibility to add autologous “cellular drugs” like modified T- or NK-cells to the mix to study their impact on the tumor tissue in a rather controlled environment. In combination with genomic data and gene expression profiles from molecular tumor boards such functional tests would greatly amplify the options to select optimal drug combinations in the personalized precision oncology setting. Thus, an ideal future experimental approach to investigate drug repurposing by tumor tissue editing could be the implementation of ‘functional/molecular tumor boards’ where patients are selected for therapies within umbrella trials not only based on molecular but also on functional tumor properties (**Figure 2**).

## Discussion

Systematic research for drug repurposing in oncology represents a wide-ranging effort considering the diversity of approaches (1–4, 7, 181–183). Here, data are summarized demonstrating that identifying tumor systems states for drug repurposing may be systematically studied combining tumor tissue editing and targeting of therapeutically edited tumor tissue with drugs, which show modest mono-activity in non-edited tumor tissue. In the first step the therapeutic approach intends to modify tumor tissue functions for tumor growth control, in the second step, to target in edited tumor tissues correspondingly altered and novel pattern of drug targets. Established experiences on two-step drug repurposing, particularly in breast cancer and multiple myeloma, but also clinical data on systematic extension of the editing technique on a broad variety of histologic different carcinomas, sarcomas and hematologic neoplasias reveal that the combinatory use of repurposed drugs for reprogramming tumor tissues may establish differential functional systems states for attenuating tumor growth, independently of the histologic tumor type (191) (4, 11, 125, 179, 192). Tissue editing is not limited to the exemplarily discussed tumor systems states, differentiation, transdifferentiation and establishing immunosurveillance and alternative tumor cell death. However, it may only be qualitatively diversified, when differential combinatory reprogramming techniques are applied. One cannot necessarily assume that all editing results exclusively fit with tumor systems states described by single pathophysiological processes, as regulatory active drug combinations simultaneously cross-interfere with multiple pathophysiological processes and hallmarks of cancer.

The therapeutic methodology provides prerequisites to systematically study drug repurposing approaches. Vice versa the therapy technique is applicable for identifying and diversifying novel combinatory drug schedules aiming at establishing novel, probably multi-layered organized tumor tissue functions attenuating tumor growth with ‘old’ drugs, or drugs in the pre-clinical developmental process (192). Commonly, drug approval is linked to monoactivity at maximal tolerable doses (43, 193). However, high clinical efficacy is often restricted to few tumor entities (12, 194). In so far, induction of anakoinosis represents a change in paradigm, and provides a novel experimental field to systematically study drug repurposing. The clinically proven activity of targeted agents, which are rather inefficacious in non-edited tumor tissue, can be interpreted with the context-dependent gain of clinical activity in edited tumor tissue, while considering tumor biology (9, 21, 26, 128). Seen from the combinatorial use of drugs an additive or synergistic drug-drug interaction may be verified. This view, however, would not differ from the classic description of the combinatorial use of any targeted therapy.

Preceding tumor tissue editing is known to impact consecutive therapies and therefore, determines therapy sequences (195). At best, tumor editing can even prolong progression-free survival, which may be translated in an overall survival benefit (128, 179, 191, 196, 197). During drug development, testing diversified systems status of tumor tissues for novel drug activities seems to be an important field for research to avoid missing significant drug activities in different tumor systems states. Importantly, such systems states may be ‘targeted’ edited (7, 9, 11, 176). The modest monoactivity of CDK4/6 inhibitors or elotuzumab in comparison to edited tumor tissue - with antihormonal therapy approaches in breast cancer and lenalidomide plus dexamethasone in multiple myeloma, respectively, - are pivotal examples (9, 126). Systematically addressing the ‘old’ but not systematically studied reservoir of drug activities for establishing biomodulatory, pro-anakoinotic therapy approaches, may close the gap that the classic targeted therapies have left behind. While proceeding with the mutation-triggered working hypothesis, the potential target reservoir of classic targeted therapies is obviously self-limiting, as those therapies do not fully take account of communicatively evolving non-cancer cell autonomous functions and structures based on phenotypic plasticity of tumor cell compartments (12). A further clinical limiting aspect ensues on the fact that also efficacy of targeted therapies may be highly context-dependent, either on different genetic or phenotypic backgrounds, as again exemplified by CDK4/6 inhibitors (126, 127).

Drug repurposing for tumor tissue editing and targeting edited tumor tissues may be applied for rescuing refractory disease, but also for the still not sufficiently studied field of chemoprevention (11). Particularly, the therapeutic technique confers possibilities to evaluate novel edited systems states for tumor control in tumor therapy or prevention (**Figure 1**).

## Author Contributions

All authors listed have made a substantial, direct, and intellectual contribution to the work and approved it for publication.

## Conflict of Interest

The authors declare that the research was conducted in the absence of any commercial or financial relationships that could be construed as a potential conflict of interest.

## Publisher’s Note

All claims expressed in this article are solely those of the authors and do not necessarily represent those of their affiliated organizations, or those of the publisher, the editors and the reviewers. Any product that may be evaluated in this article, or claim that may be made by its manufacturer, is not guaranteed or endorsed by the publisher.
